# Preoperative Imaging for Nodal Assessment in Endometrial Carcinoma: A Comparative Study of MRI and 18F-FDG PET/CT Across Risk Groups

**DOI:** 10.3390/medicina62071418

**Published:** 2026-07-22

**Authors:** Süleyman Özen, Eda Güner Özen, Muzaffer Sancı

**Affiliations:** 1 Department of Gynecologic Oncology, İzmir City Hospital, İzmir 35540, Turkey; 2 Department of Obstetrics and Gynecology, İzmir City Hospital, İzmir 35540, Turkey

**Keywords:** endometrial carcinoma, lymph node metastasis, magnetic resonance imaging, 18F-FDG PET/CT, preoperative staging, sentinel lymph node, risk stratification, gynecologic oncology

## Abstract

*Background and Objectives*: Accurate preoperative assessment of lymph node metastasis is essential for surgical staging and treatment planning in endometrial carcinoma. Magnetic resonance imaging (MRI) is widely used for local staging; however, its nodal performance is limited by reliance on morphologic criteria. 18F-FDG PET/CT provides whole-body functional assessment and may support decision-making when sentinel lymph node (SLN) mapping is unavailable. This study compared MRI and 18F-FDG PET/CT for preoperative detection of pelvic and paraaortic nodal metastases across pragmatic histologic risk groups. *Materials and Methods*: This retrospective diagnostic-accuracy study included 255 consecutive surgically treated patients with histologically confirmed endometrial carcinoma who underwent both preoperative pelvic MRI and whole-body 18F-FDG PET/CT at a tertiary gynecologic oncology center between December 2023 and December 2025. All patients underwent pelvic lymphadenectomy, 111 patients (43.5%) also underwent paraaortic lymphadenectomy, and SLN mapping was not performed. Patients were categorized as Group 1 (Grade 1–2 endometrioid adenocarcinoma) or Group 2 (Grade 3 endometrioid or non-endometrioid/aggressive histology). Diagnostic estimates were reported with 95% confidence intervals, paired comparisons were performed using exact McNemar tests, and multivariable bias-reduced logistic regression was used to assess independent imaging associations with nodal metastasis. *Results*: Any nodal metastasis was documented in 24 patients (9.4%). 18F-FDG PET/CT showed higher sensitivity than MRI for patient-level nodal detection (91.7%, 95% CI 74.2–97.7 vs. 37.5%, 95% CI 21.2–57.3), with similarly high specificity (97.0%, 95% CI 93.9–98.5 vs. 97.8%, 95% CI 95.0–99.1). The NPV of 18F-FDG PET/CT was 99.1% (95% CI 96.8–99.8), compared with 93.8% (95% CI 90.0–96.2) for MRI. MRI sensitivity was particularly low in Group 1 (18.2%, 95% CI 5.1–47.7), whereas PET/CT retained high sensitivity in both groups, though the perfect performance observed in Group 2 had wide confidence limits. *Conclusions*: 18F-FDG PET/CT demonstrated superior sensitivity and NPV compared with MRI for preoperative nodal assessment in endometrial carcinoma. These results support the selective use of PET/CT as an adjunctive staging tool in settings without SLN mapping, but they should not be interpreted as evidence that PET/CT can replace histologic nodal staging.

## 1. Introduction

Endometrial carcinoma (EC) is the most common gynecologic malignancy in developed countries and represents an increasing component of the global cancer burden. GLOBOCAN 2022 estimates indicate more than 420,000 new cases of cancers of the corpus uteri and nearly 100,000 deaths worldwide each year, with the highest incidence rates reported in regions with high or very high Human Development Index [1]. This epidemiologic increase has been associated with population aging, obesity, metabolic disease, and shifting reproductive patterns [2]. Although most patients present with uterine-confined disease, lymph node metastasis (LNM) remains one of the strongest prognostic factors and directly affects adjuvant treatment recommendations, recurrence risk, and survival outcomes [3].

Accurate nodal assessment is therefore central to modern EC management. Historically, systematic pelvic and paraaortic lymphadenectomy provided the surgical reference standard for nodal staging. However, routine lymphadenectomy increases operative time and perioperative morbidity and is associated with long-term complications such as lower-extremity lymphedema, without a clear survival advantage in patients with low-risk uterine features [4]. This has driven a shift toward risk-adapted staging strategies that integrate preoperative histology, imaging, intraoperative findings, and SLN mapping.

Preoperative imaging has an important role in this individualized pathway. Pelvic MRI is the standard imaging modality for local staging because it provides high soft-tissue contrast and supports assessment of myometrial invasion, cervical stromal invasion, and extrauterine extension [5]. Nevertheless, MRI nodal assessment depends mainly on size, morphology, diffusion restriction, and enhancement patterns; consequently, small-volume nodal metastases within normal-sized lymph nodes may be missed. In contrast, 18F-FDG PET/CT combines anatomic and metabolic information and provides whole-body assessment, including pelvic, paraaortic, and distant disease sites.

Current ESGO-ESTRO-ESP guidance and the FIGO 2023 staging system increasingly emphasize integrated histologic and molecular risk stratification, including molecular class where available, lymphovascular space invasion, and histologic subtype [6,7]. In many retrospective cohorts, however, universal molecular classification is not yet available. For this reason, studies evaluating imaging in routine surgical practice must clearly distinguish between guideline-defined molecular risk groups and histology-based study categories. In the present study, the terms Group 1 and Group 2 refer to histologic risk strata only, not to complete ESGO molecular risk groups.

Although several studies and meta-analyses suggest that PET-based imaging improves nodal detection compared with anatomic imaging alone, reported performance varies substantially according to prevalence of nodal disease, histologic risk distribution, reference standards, and imaging interpretation criteria. Moreover, many centers still lack near-infrared fluorescence infrastructure for indocyanine green-guided SLN mapping. The present study was therefore designed to compare preoperative MRI and 18F-FDG PET/CT for nodal assessment in a representative tertiary-center cohort of patients with EC. We focused on diagnostic performance across histologic risk groups, quantified uncertainty with confidence intervals, and explicitly addressed verification bias, false-positive and false-negative findings, and the limitations of imaging-based staging.

## 2. Materials and Methods

### 2.1. Study Design, Setting, and Reporting Framework

This retrospective observational diagnostic-accuracy study was conducted at İzmir City Hospital, Department of Gynecologic Oncology Surgery. The study included patients diagnosed with EC who were discussed at the institutional tumor board between December 2023 and December 2025 and for whom primary surgical treatment was recommended. The study was approved by the Institutional Review Board of İzmir City Hospital (decision number 2026/1; 7 January 2026). The manuscript was revised in accordance with STROBE principles for observational studies and STARD principles for diagnostic-accuracy reporting [8,9].

**Figure 1:**
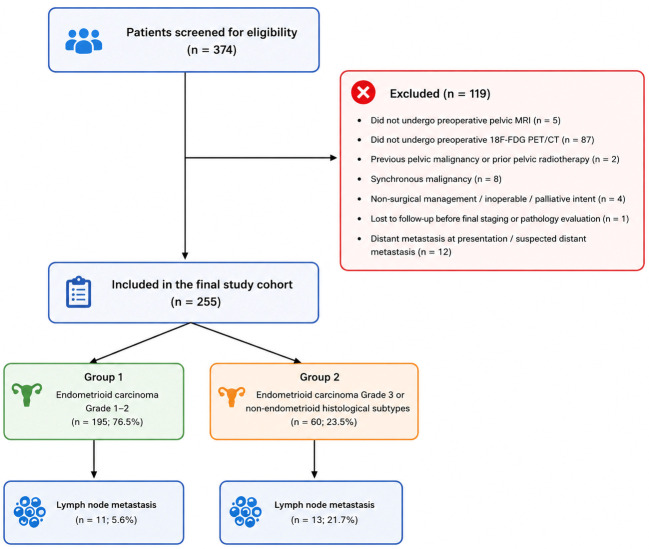
STROBE-style patient flow diagram showing screening, exclusion criteria, eligibility assessment, final inclusion, and stratification by histologic risk group.

The patient-selection process is summarized in Figure 1.

### 2.2. Eligibility Criteria

Patients were included if they had a histopathologically confirmed diagnosis of EC, underwent both preoperative pelvic MRI and whole-body 18F-FDG PET/CT at our institution within four weeks before surgery, were treated surgically by a gynecologic oncologist, and had complete clinicopathologic data available for final histopathologic staging. Patients were excluded if either imaging modality was absent or technically incomplete, if final pathology or imaging reports were incomplete, if there was a history of pelvic malignancy or pelvic radiotherapy, if synchronous malignancy was diagnosed, if distant metastasis was confirmed before surgery and the patient was managed with palliative or non-surgical intent, or if the patient was lost before final pathological assessment. No patient in the final analytic cohort was classified as FIGO stage IV.

### 2.3. Histologic Risk Grouping

For subgroup analysis, patients were stratified into two histology-based risk categories. Group 1 included patients with Grade 1–2 endometrioid adenocarcinoma. Group 2 included patients with Grade 3 endometrioid adenocarcinoma, serous carcinoma, clear cell carcinoma, carcinosarcoma, mixed serous/clear-cell carcinoma, or other pathology-report wording that did not meet the strict definition of low-grade endometrioid adenocarcinoma. This classification was chosen to reflect preoperative histology-driven clinical decision-making in a setting where universal molecular classification was not available. We therefore do not claim that Group 1 and Group 2 reproduce the full ESGO-ESTRO-ESP molecular risk groups [6]. This limitation is explicitly addressed in the Discussion.

### 2.4. Imaging Protocols and Interpretation

All included patients underwent pelvic MRI and whole-body 18F-FDG PET/CT as part of routine preoperative staging. MRI examinations were performed on a 1.5-T Siemens^®^ MAGNETOM Aera system (Siemens^®^, Erlangen, Germany) using a dedicated pelvic phased-array coil. The protocol included axial and sagittal T2-weighted sequences, axial T1-weighted sequences, diffusion-weighted imaging with apparent diffusion coefficient maps, and dynamic contrast-enhanced sequences when clinically appropriate. MRI was used to assess myometrial invasion, cervical stromal invasion, adnexal or parametrial extension, and suspicious nodal disease.

MRI lymph node positivity was defined prospectively from clinical reports using standardized morphologic and functional criteria. A lymph node was considered suspicious if it had a short-axis diameter ≥10 mm in pelvic or paraaortic basins, round configuration, irregular border, loss of fatty hilum, necrotic or heterogeneous internal signal, abnormal enhancement, or restricted diffusion on DWI/ADC maps. Nodes that were borderline in size but demonstrated restricted diffusion or clearly abnormal morphology were considered positive.

18F-FDG PET/CT images were acquired using a Philips^®^ Gemini TF 16-slice PET/CT scanner (Philips^®^ Healthcare, Cleveland, OH, USA) according to institutional protocol after fasting for at least 6 h. Blood glucose was verified to be <150 mg/dL before tracer injection, and imaging began approximately 60 min after 18F-FDG administration. The acquisition field extended from the skull base to mid-thigh. PET/CT was interpreted visually by nuclear medicine physicians as part of routine care. A lymph node was considered positive when focal FDG uptake exceeded regional background activity and corresponded anatomically to an expected pelvic or paraaortic nodal basin, regardless of nodal size. SUVmax was recorded when available in clinical reports, but no pre-specified SUVmax threshold was used as a mandatory positivity criterion; therefore, a post hoc SUVmax threshold analysis was not performed.

MRI and PET/CT reports were issued before surgery and before final pathological staging. In routine clinical workflow, MRI and PET/CT were interpreted by different specialists. Final diagnostic classification was confirmed at the multidisciplinary tumor board. Formal interobserver agreement was not calculated because the retrospective dataset relied on final clinical reports rather than a protocolized double-read by multiple blinded readers; this is acknowledged as a reproducibility limitation.

### 2.5. Surgical and Pathological Evaluation

All patients underwent total hysterectomy with bilateral salpingo-oophorectomy. No patient underwent SLN mapping in this cohort because the required near-infrared fluorescence infrastructure for indocyanine green-guided mapping was not available during the study period. Accordingly, SLN technique, mapping rate, ultrastaging, and management of mapping failure were not applicable. Omentectomy was routinely performed in serous carcinoma and carcinosarcoma and selectively in other aggressive histologies according to oncologic practice. Final pathology included histologic subtype, tumor grade, depth of myometrial invasion, lymphovascular space invasion (LVSI), lymph node metastasis, hormone receptor status, p53 expression, and final FIGO stage according to the 2009 staging criteria.

Histological nodal assessment was based on lymphadenectomy specimens. Pelvic lymphadenectomy was performed in all 255 patients. Paraaortic lymphadenectomy was performed in 111 patients (43.5%), including all Group 2 patients and Group 1 patients with deep myometrial invasion. Paraaortic lymphadenectomy was omitted in 144 low-grade endometrioid patients without deep myometrial invasion (56.5%). Thus, 111 patients underwent both pelvic and paraaortic lymphadenectomy, 144 underwent pelvic lymphadenectomy only, and no patient had no histological nodal assessment. The patient-level reference standard for any nodal metastasis was based on histological evaluation of surgically removed pelvic nodes and, when available, paraaortic nodes. In patients without paraaortic lymphadenectomy, patient-level node-negative status required negative pelvic node histology together with absence of suspicious paraaortic disease on preoperative MRI/PET/CT, intraoperative assessment, final surgical-pathological review, and multidisciplinary tumor board evaluation. Region-level pelvic analyses included all surgically assessed pelvic basins (*n* = 255), whereas region-level paraaortic analyses were restricted to histologically assessed paraaortic basins (*n* = 111). This selective paraaortic reference standard may affect estimates of specificity and particularly NPV; this limitation is emphasized in the Discussion.

### 2.6. FIGO 2009 and FIGO 2023 Considerations

Final staging in the analytic dataset was recorded using FIGO 2009 criteria because this was the institutional staging format used in the operative-pathology records during data extraction [10]. The FIGO 2023 system incorporates molecular classification, histologic subtype, and more granular prognostic categories [7]. Complete FIGO 2023 restaging was not feasible because POLE mutation and mismatch repair status were not uniformly available. Nevertheless, the imaging endpoint of this study—presence or absence of pelvic/paraaortic nodal metastasis—would remain clinically relevant under both systems. We therefore report the original FIGO 2009 stage distribution and discuss how the findings should be interpreted under contemporary FIGO 2023 and ESGO-ESTRO-ESP frameworks.

### 2.7. Statistical Analysis

Statistical analysis was performed using IBM SPSS Statistics version 26.0. Continuous variables were assessed for normality and reported as mean ± standard deviation. Between-group comparisons used Student’s *t*-test or the Mann–Whitney U test when distributional assumptions were not met. Categorical variables were summarized as counts and percentages and compared using Fisher’s exact test or the chi-square test as appropriate.

Diagnostic performance was calculated from 2 × 2 tables using the nodal reference standard described above. For patient-level analyses, any pelvic or paraaortic nodal metastasis was considered positive. For region-level analyses, pelvic estimates were calculated in all 255 patients who underwent pelvic lymphadenectomy, whereas paraaortic estimates were calculated only in the 111 patients who underwent paraaortic lymphadenectomy; patients without histological paraaortic assessment were not included in paraaortic region-level specificity, NPV, or accuracy denominators. Sensitivity, specificity, positive predictive value (PPV), negative predictive value (NPV), and accuracy are reported with 95% confidence intervals. Wilson score confidence intervals were used for proportions. Paired comparisons of sensitivity and specificity between MRI and 18F-FDG PET/CT were performed using exact McNemar tests among node-positive and node-negative patients, respectively. Because multiple subgroup comparisons were exploratory, Holm-adjusted *p*-values were calculated as a sensitivity check; both unadjusted and adjusted interpretations are reported. Receiver operating characteristic analysis was not performed because both imaging modalities were evaluated as binary index tests rather than continuous threshold-based measurements.

A multivariable analysis was added using bias-reduced Firth logistic regression because conventional logistic regression showed quasi-complete separation for some predictors in the presence of only 24 node-positive events. The model included PET/CT positivity, MRI positivity, histologic risk group, and LVSI. Deep myometrial invasion was not included in the final multivariable model because all node-positive patients in the analytic dataset had deep myometrial invasion, producing complete separation. A two-sided *p*-value < 0.05 was considered statistically significant.

## 3. Results

### 3.1. Study Population

The final cohort included 255 surgically treated patients with histologically confirmed EC. Group 1 comprised 195 patients with Grade 1–2 endometrioid adenocarcinoma, and Group 2 comprised 60 patients with Grade 3 endometrioid or non-endometrioid/aggressive histology. Demographic and clinicopathologic characteristics are summarized in Table 1. Group 2 patients were older than Group 1 patients (65.03 ± 9.15 vs. 60.76 ± 9.66 years; *p* = 0.002). BMI did not differ significantly between groups. Deep myometrial invasion, LVSI, lymph node metastasis, hormone receptor loss, and p53 positivity/aberrant expression were more frequent in Group 2.

### 3.2. FIGO Stage and Histological Subtypes

The distribution of FIGO 2009 stage and histological subtype is shown in Table 2. No included patient had FIGO stage IV disease. Stage IIIC disease was documented in 24 patients (9.4%), including 3 with IIIC1 and 21 with IIIC2 disease. Histology was significantly different between groups by design, with Group 2 containing Grade 3 endometrioid and non-endometrioid/aggressive histologies.

### 3.3. Surgical Nodal Assessment and Reference Standard

Details of the surgical nodal assessment are presented in Table 3. No SLN mapping was attempted, and no SLN ultrastaging was performed. Pelvic lymphadenectomy was performed in all 255 patients. Paraaortic lymphadenectomy was performed in 111 patients (43.5%); 144 patients (56.5%) underwent pelvic lymphadenectomy only because they had low-grade endometrioid carcinoma without deep myometrial invasion. No patient had no histological nodal assessment. Patient-level node-negative status in patients without paraaortic lymphadenectomy was assigned only when pelvic nodes were pathologically negative and no paraaortic disease was suspected on preoperative imaging, intraoperative evaluation, final pathology review, or multidisciplinary tumor board assessment.

### 3.4. Patient-Level Diagnostic Performance

Any lymph node metastasis was documented in 24 patients (9.4%). Patient-level diagnostic performance is presented in Table 4. In the overall cohort, 18F-FDG PET/CT detected 22 of 24 node-positive patients, whereas MRI detected 9 of 24. 18F-FDG PET/CT therefore had substantially higher sensitivity than MRI (91.7%, 95% CI 74.2–97.7 vs. 37.5%, 95% CI 21.2–57.3; exact McNemar *p* = 0.002; Holm-adjusted *p* = 0.021). Specificity was high for both modalities and did not differ significantly (97.0%, 95% CI 93.9–98.5 vs. 97.8%, 95% CI 95.0–99.1; exact McNemar *p* = 0.774).

In Group 1, MRI sensitivity was 18.2% (95% CI 5.1–47.7), corresponding to only 2 true-positive MRI findings among 11 node-positive patients. The PPV of 50.0% in Group 1 was based on very small numbers (2 true positives and 2 false positives) and had a wide confidence interval (15.0–85.0), indicating substantial statistical uncertainty. In Group 2, PET/CT showed perfect point estimates; however, these estimates were derived from only 60 patients and 13 node-positive cases, and the confidence intervals remain wide. These subgroup findings are best viewed as exploratory.

### 3.5. Region-Level Pelvic and Paraaortic Performance

Pelvic and paraaortic nodal involvement was identified in 23 and 8 histologically assessed nodal regions, respectively; because some patients had both pelvic and paraaortic involvement, the total number of involved nodal regions exceeded the number of unique node-positive patients. Region-level diagnostic estimates are shown in Table 5. Pelvic estimates were based on all 255 patients with pelvic lymphadenectomy, whereas paraaortic estimates were restricted to the 111 patients with paraaortic lymphadenectomy. 18F-FDG PET/CT had higher sensitivity than MRI for pelvic regions (87.0% vs. 43.5%) and retained high specificity in both pelvic and paraaortic nodal basins.

### 3.6. False-Positive and False-Negative Findings

Discordant findings were analyzed descriptively. 18F-FDG PET/CT produced 7 false-positive and 2 false-negative patient-level results. All PET/CT false-positive cases occurred in Group 1 and were stage I on final pathology; all had deep myometrial invasion but no LVSI, suggesting that reactive or inflammatory nodal FDG uptake may have contributed to false positivity. The 2 PET/CT false-negative cases were both Group 1 patients with final stage IIIC2 disease and deep myometrial invasion, consistent with small-volume or metabolically low-level nodal disease below PET/CT detection limits. MRI produced 5 false-positive and 15 false-negative patient-level results. All MRI false-negative cases had deep myometrial invasion, and most were stage IIIC2, supporting the known limitation of size- and morphology-based MRI criteria in normal-sized metastatic nodes.

### 3.7. Multivariable Analysis

Bias-reduced multivariable logistic regression is shown in Table 6. PET/CT positivity and MRI positivity were both independently associated with nodal metastasis after adjustment for histologic risk group and LVSI. The magnitude of the adjusted ORs was large, reflecting the low prevalence of nodal metastasis and quasi-separation in the dataset; therefore, the estimates should be interpreted primarily as evidence of independent association rather than as precise effect-size estimates.

## 4. Discussion

In this retrospective diagnostic-accuracy study of 255 patients with surgically treated EC, 18F-FDG PET/CT demonstrated substantially higher sensitivity and NPV than MRI for preoperative detection of lymph node metastasis, while both modalities maintained high specificity. The principal clinical implication is not that PET/CT can replace surgical nodal staging, but that PET/CT may provide additional noninvasive information for risk-adapted preoperative planning, particularly in centers where SLN mapping is not routinely available.

Our findings are consistent with prior systematic reviews and imaging-focused studies showing that PET-based imaging generally provides higher sensitivity and overall accuracy for nodal metastasis than anatomic techniques alone [11,12,13,14,15,16]. MRI remains essential for local uterine staging, including assessment of myometrial invasion, cervical stromal invasion, and extrauterine pelvic extension [17]. However, MRI nodal staging is intrinsically limited by its dependence on size and morphology. This limitation was evident in Group 1, in which MRI sensitivity was only 18.2%. The very wide CI around the Group 1 MRI PPV confirms that the PPV estimate is unstable and should not be overinterpreted.

The unexpectedly perfect point estimates for PET/CT in Group 2 require careful interpretation. Although all 13 node-positive Group 2 patients were PET/CT-positive and no PET/CT false-positive results were observed in this subgroup, the confidence intervals remain wide because the subgroup included only 60 patients. This finding may reflect a higher burden of metabolically active disease in aggressive histologies, but it may also reflect the small number of events and the partial verification structure of the cohort. The manuscript therefore avoids claiming that PET/CT provides definitive nodal staging in high-risk EC.

False-positive and false-negative findings provide clinically relevant insight. PET/CT false positives were confined to low-grade endometrioid cases and may plausibly reflect inflammatory or reactive nodes. Conversely, PET/CT false negatives occurred in final IIIC2 disease, consistent with the limited spatial resolution of PET/CT for small-volume metastases. MRI false negatives were more frequent and likely reflect metastases in normal-sized nodes. These findings support using PET/CT as an adjunctive triage tool rather than as a standalone substitute for histologic nodal assessment.

SLN mapping remains the preferred contemporary surgical staging strategy when the required infrastructure and expertise are available. The FIRES, SENTOR, and SHREC studies and high-grade EC meta-analyses report high sensitivity and NPV for SLN mapping with substantially lower morbidity than systematic lymphadenectomy [18,19,20,21,22]. Therefore, the imaging-based context of this study should not be interpreted as a direct comparison between PET/CT and SLN mapping. Rather, the study addresses a practical clinical scenario: how preoperative imaging performs when SLN mapping is not routinely available.

The FIGO 2023 and ESGO-ESTRO-ESP frameworks place nodal status within a broader biologic context that includes histologic subtype and molecular classification [6,7]. Our dataset was staged according to FIGO 2009 and lacked uniform POLE and mismatch repair data; consequently, full molecular restaging was not possible. Nevertheless, the primary endpoint—whether preoperative imaging detects nodal metastasis—remains relevant under FIGO 2023 because nodal involvement continues to guide surgical and adjuvant management. Future studies should prospectively evaluate PET/CT and MRI within fully molecularly classified cohorts [23].

The clinical utility of PET/CT also depends on resource availability, cost, and downstream consequences. PET/CT may detect unsuspected extrauterine or distant disease and may guide the extent of nodal dissection; however, it is more expensive than MRI in many systems and may generate false-positive findings that prompt additional surgery or imaging. Cost-effectiveness analyses currently support SLN mapping as a dominant or cost-effective strategy compared with lymphadenectomy in high-risk EC [24]. PET/CT should therefore be framed as an adjunct in selected settings rather than as a cost-equivalent alternative to SLN mapping.

This study has several limitations. First, the retrospective single-center design introduces selection bias. Second, although all patients underwent pelvic lymphadenectomy, paraaortic lymphadenectomy was omitted in 144 low-grade endometrioid patients without deep myometrial invasion. Patient-level node-negative status in these patients therefore relied on negative pelvic node histology together with negative paraaortic imaging, intraoperative assessment, final pathology review, and multidisciplinary tumor board evaluation rather than paraaortic histology. This selective paraaortic reference standard may overestimate NPV for paraaortic disease and for patient-level any nodal metastasis in low-risk patients. Third, the number of node-positive patients was low, leading to wide confidence intervals and unstable subgroup estimates. Fourth, imaging interpretation was based on routine clinical reports rather than centralized blinded rereading, preventing interobserver agreement analysis. Fifth, PET/CT interpretation was visual, and SUVmax thresholds were not available for standardized post hoc analysis. Finally, complete molecular classification was not uniformly available, limiting direct application of contemporary ESGO molecular risk groups and FIGO 2023 restaging.

Despite these limitations, the study contributes a comparatively large single-center cohort in which every included patient underwent both MRI and 18F-FDG PET/CT before primary surgery and at least pelvic histological nodal assessment. By adding confidence intervals, paired statistical comparisons, multivariable modeling, discordant-case analysis, and explicit clarification of the nodal reference standard, the analysis provides a more balanced interpretation of PET/CT performance.

## 5. Conclusions

18F-FDG PET/CT demonstrated higher sensitivity and NPV than MRI for preoperative detection of nodal metastasis in EC, while both modalities had high specificity. The results suggest that PET/CT can support preoperative risk-adapted planning where SLN mapping is unavailable, but it should not be considered a replacement for histologic nodal staging. Prospective multicenter studies with standardized imaging criteria, complete molecular classification, uniform nodal reference standards, and formal cost-effectiveness evaluation are needed.

## Figures and Tables

**Table 1:** Demographic and clinicopathologic characteristics of the study population.

Variable	Overall (*n* = 255)	Group 1 (*n* = 195)	Group 2 (*n* = 60)	*p*-Value
Age, mean ± SD (years)	61.76 ± 9.69	60.76 ± 9.66	65.03 ± 9.15	0.002
BMI, mean ± SD (kg/m^2^)	27.82 ± 2.46	27.85 ± 2.42	27.70 ± 2.59	0.689
Deep myometrial invasion (≥50%)	87/255 (34.1%)	51/195 (26.2%)	36/60 (60.0%)	<0.001
LVSI	34/255 (13.3%)	20/195 (10.3%)	14/60 (23.3%)	0.015
Lymph node metastasis	24/255 (9.4%)	11/195 (5.6%)	13/60 (21.7%)	<0.001
Hormone receptor loss	81/255 (31.8%)	52/195 (26.7%)	29/60 (48.3%)	0.002
p53 positivity/aberrant expression	102/255 (40.0%)	70/195 (35.9%)	32/60 (53.3%)	0.023

BMI, body mass index; LVSI, lymphovascular space invasion. Hormone receptor loss and p53 positivity/aberrant expression were coded according to the final pathology report available in the analytic dataset.

**Table 2:** FIGO 2009 stage and histological subtype distribution.

Variable	Overall (*n* = 255)	Group 1 (*n* = 195)	Group 2 (*n* = 60)	*p*-Value
FIGO 2009 stage				<0.001
Stage IA	161/255 (63.1%)	147/195 (75.4%)	14/60 (23.3%)	
Stage IB	46/255 (18.0%)	33/195 (16.9%)	13/60 (21.7%)	
Stage II	22/255 (8.6%)	2/195 (1.0%)	20/60 (33.3%)	
Stage IIIA/B	2/255 (0.8%)	2/195 (1.0%)	0/60 (0.0%)	
Stage IIIC1	3/255 (1.2%)	0/195 (0.0%)	3/60 (5.0%)	
Stage IIIC2	21/255 (8.2%)	11/195 (5.6%)	10/60 (16.7%)	
Stage IV	0/255 (0.0%)	0/195 (0.0%)	0/60 (0.0%)	
Histological subtype/risk category				<0.001
Endometrioid adenocarcinoma, Grade 1–2	195/255 (76.5%)	195/195 (100.0%)	0/60 (0.0%)	
Endometrioid adenocarcinoma, Grade 3	13/255 (5.1%)	0/195 (0.0%)	13/60 (21.7%)	
Serous carcinoma	14/255 (5.5%)	0/195 (0.0%)	14/60 (23.3%)	
Clear cell carcinoma	11/255 (4.3%)	0/195 (0.0%)	11/60 (18.3%)	
Carcinosarcoma	9/255 (3.5%)	0/195 (0.0%)	9/60 (15.0%)	
Mixed serous/clear-cell carcinoma	7/255 (2.7%)	0/195 (0.0%)	7/60 (11.7%)	
Other high-risk/ambiguous endometrial carcinoma	6/255 (2.4%)	0/195 (0.0%)	6/60 (10.0%)	

Histologic subtype wording was harmonized from pathology reports. The grouping is histology-based and does not represent complete ESGO molecular risk classification.

**Table 3:** Surgical nodal assessment and nodal reference standard.

Variable	*n*/*N* (%) or Description
SLN mapping attempted	0/255 (0.0%)
SLN mapping technique, mapping rate, mapping failure management, and ultrastaging	Not applicable; SLN mapping was not performed
Pelvic lymphadenectomy	255/255 (100.0%)
Paraaortic lymphadenectomy	111/255 (43.5%)
Pelvic and paraaortic lymphadenectomy	111/255 (43.5%)
Pelvic lymphadenectomy only	144/255 (56.5%)
No histological nodal assessment	0/255 (0.0%)
Region-level pelvic analysis denominator	255 surgically assessed pelvic basins
Region-level paraaortic analysis denominator	111 surgically assessed paraaortic basins

SLN, sentinel lymph node. Group 1 patients without deep myometrial invasion underwent pelvic lymphadenectomy without paraaortic lymphadenectomy according to institutional surgical practice.

**Table 4:** Patient-level diagnostic performance of MRI and 18F-FDG PET/CT for lymph node metastasis.

Imaging Modality	Subgroup	Sensitivity % (95% CI)	Specificity % (95% CI)	PPV % (95% CI)	NPV % (95% CI)	Accuracy % (95% CI)
18F-FDGPET/CT	Overall	91.7 (74.2–97.7)	97.0 (93.9–98.5)	75.9 (57.9–87.8)	99.1 (96.8–99.8)	96.5 (93.4–98.1)
18F-FDG PET/CT	Group 1	81.8 (52.3–94.9)	96.2 (92.4–98.1)	56.2 (33.2–76.9)	98.9 (96.0–99.7)	95.4 (91.5–97.6)
18F-FDG PET/CT	Group 2	100.0 (77.2–100.0)	100.0 (92.4–100.0)	100.0 (77.2–100.0)	100.0 (92.4–100.0)	100.0 (94.0–100.0)
MRI	Overall	37.5 (21.2–57.3)	97.8 (95.0–99.1)	64.3 (38.8–83.7)	93.8 (90.0–96.2)	92.2 (88.2–94.9)
MRI	Group 1	18.2 (5.1–47.7)	98.9 (96.1–99.7)	50.0 (15.0–85.0)	95.3 (91.3–97.5)	94.4 (90.2–96.8)
MRI	Group 2	53.8 (29.1–76.8)	93.6 (82.8–97.8)	70.0 (39.7–89.2)	88.0 (76.2–94.4)	85.0 (73.9–91.9)

Values are percentages with 95% confidence intervals unless otherwise indicated. PET/CT, positron emission tomography/computed tomography; PPV, positive predictive value; NPV, negative predictive value.

**Table 5:** Region-level diagnostic performance of MRI and 18F-FDG PET/CT for pelvic and paraaortic nodal metastases.

Imaging Modality	Region	Sensitivity % (95% CI)	Specificity % (95% CI)	PPV % (95% CI)	NPV % (95% CI)	Accuracy % (95% CI)
18F-FDG PET/CT	Pelvic	87.0 (67.9–95.5)	99.1 (96.9–99.8)	90.9 (72.2–97.5)	98.7 (96.3–99.6)	98.0 (95.5–99.2)
18F-FDG PET/CT	Paraaortic	87.5 (52.9–97.8)	99.0 (94.7–99.8)	87.5 (52.9–97.8)	99.0 (94.7–99.8)	98.2 (93.7–99.5)
MRI	Pelvic	43.5 (25.6–63.2)	98.7 (96.3–99.6)	76.9 (49.7–91.8)	94.6 (91.0–96.8)	93.7 (90.1–96.1)
MRI	Paraaortic	50.0 (21.5–78.5)	98.1 (93.2–99.5)	66.7 (30.0–90.3)	96.2 (90.6–98.5)	94.6 (88.7–97.5)

Values are percentages with 95% confidence intervals unless otherwise indicated. Pelvic estimates were calculated in all 255 patients with pelvic lymphadenectomy; paraaortic estimates were restricted to the 111 patients with paraaortic lymphadenectomy. PET/CT, positron emission tomography/computed tomography; PPV, positive predictive value; NPV, negative predictive value.

**Table 6:** Bias-reduced multivariable logistic regression for lymph node metastasis.

Variable	Adjusted OR	95% CI	*p*-Value
18F-FDG PET/CT positive	691.81	48.38–9891.63	<0.001
MRI positive	167.85	8.62–3269.45	<0.001
Group 2 histologic risk	1.61	0.26–10.00	0.609
LVSI	4.32	0.59–31.47	0.149

The model included 255 patients and 24 node-positive events. Deep myometrial invasion was excluded because all node-positive patients had deep myometrial invasion, producing complete separation. OR, odds ratio; LVSI, lymphovascular space invasion.

## Data Availability

The datasets generated and/or analyzed during the current study are not publicly available due to institutional data protection regulations and patient confidentiality requirements but are available from the corresponding author upon reasonable request.

## Abbreviations

EC, endometrial carcinoma; LNM, lymph node metastasis; MRI, magnetic resonance imaging; PET/CT, positron emission tomography/computed tomography; FDG, fluorodeoxyglucose; SLN, sentinel lymph node; FIGO, International Federation of Gynecology and Obstetrics; LVSI, lymphovascular space invasion; PPV, positive predictive value; NPV, negative predictive value.
